# Biochemical and Transcriptional Responses in Cold-Acclimated and Non-Acclimated Contrasting Camelina Biotypes under Freezing Stress

**DOI:** 10.3390/plants11223178

**Published:** 2022-11-21

**Authors:** Jahad Soorni, Seyed Kamal Kazemitabar, Danial Kahrizi, Ali Dehestani, Nadali Bagheri, Attila Kiss, Péter Gergő Kovács, István Papp, Iman Mirmazloum

**Affiliations:** 1 Department of Plant Breeding and Biotechnology, Sari Agricultural Sciences and Natural Resources University (SANRU), Sari 68984, Iran; 2 Genetics and Agricultural Biotechnology Institute of Tabarestan (GABIT), Sari Agricultural Sciences and Natural Resources University, Sari 68984, Iran; 3 Department of Agronomy and Plant Breeding, Faculty of Agriculture, Razi University, Kermanshah 67144, Iran; 4 Agro-Food Science Techtransfer and Innovation Centre, Faculty for Agro-, Food- and Environmental Science, Debrecen University, H-4032 Debrecen, Hungary; 5 Department of Agronomy, Institute of Agronomy, Hungarian University of Agriculture and Life Sciences, 2100 Gödöllő, Hungary; 6 Department of Plant Physiology and Plant Ecology, Institute of Agronomy, Hungarian University of Agriculture and Life Sciences, 1118 Budapest, Hungary

**Keywords:** cold acclimation, freezing tolerance, *C. sativa*, electrolyte leakage, gene expression

## Abstract

Cold-acclimated and non-acclimated contrasting Camelina (*Camelina sativa* L.) biotypes were investigated for changes in stress-associated biomarkers, including antioxidant enzyme activity, lipid peroxidation, protein, and proline content. In addition, a well-known freezing tolerance pathway participant known as C-repeat/DRE-binding factors (CBFs), an inducer of CBF expression (ICE1), and a cold-regulated (*COR6.6*) genes of the ICE-CBF-COR pathway were studied at the transcriptional level on the doubled-haploid (DH) lines. Freezing stress had significant effects on all studied parameters. The cold-acclimated DH34 (a freezing-tolerant line) showed an overall better performance under freezing stress than non-acclimated plants. The non-cold-acclimated DH08 (a frost-sensitive line) showed the highest electrolyte leakage after freezing stress. The highest activity of antioxidant enzymes (glutathione peroxidase, superoxide dismutase, and catalase) was also detected in non-acclimated plants, whereas the cold-acclimated plants showed lower enzyme activities upon stress treatment. Cold acclimation had a significantly positive effect on the total protein and proline content of stressed plants. The qRT-PCR analysis revealed significant differences in the expression and cold-inducibility of *CsCBF1-3*, *CsICE1*, and *CsCOR6.6* genes among the samples of different treatments. The highest expression of all CBF genes was recorded in the non-acclimated frost-tolerant biotype after freezing stress. Interestingly a significantly higher expression of *COR6.6* was detected in cold-acclimated samples of both frost-sensitive and -tolerant biotypes after freezing stress. The presented results provide more insights into freezing tolerance mechanisms in the Camelina plant from both a biochemical point of view and the expression of the associated genes.

## 1. Introduction

Camelina, also known as Siberian oilseed, is an emerging oilseed crop with remarkable constituents and agronomical advantages [1,2]. Enhancing the abiotic stress tolerance in camelina is now the subject of intensive breeding programs to identify the tolerant cultivars with increased yield and productivity [3,4,5].

Plants respond to low temperatures and frost by adopting various mechanisms to cope with or combat stress. With this regard, understanding the plant’s molecular (transcriptome) and physiological (e.g., antioxidant defense system) processes in response to low-temperature stress provides new opportunities for crop breeding to address these types of stresses [6,7].

Recent experimental reports have provided extensive information on different genes and their associated systems for low-temperature-stress tolerance in plants [8,9]. Many studies have been conducted on various factors affecting the networks responsible for tolerating low temperatures, and especially in Arabidopsis, which is a very close relative to Camelina.

The main cold-responsive signaling pathway in plants is known as the ICE-CBF-COR cascade, which consists of several genes in plant species [10]. The cold-activated ICE genes induce and regulate the expression of the C-repeat binding factor (CBF) in plants. The Arabidopsis genome encodes six paralogs of *CBF*s, with their most important three transcription factors, including *CBF1*/*DREB1B*, *CBF2*/*DREB1C*, and *CBF3*/*DREB1A*, being responsible for tolerating low temperatures [11]. All three CBF genes are parallel to each other and have high sequence similarity [12]. *CBF*/*DREB1* transcription factors have been reported to play a key role in modulating the response to cold stress in Arabidopsis and have been tested to increase cold resistance in susceptible plants [13]. Overexpression of Arabidopsis CBF genes in *B. napus* induced the expression of CBF target genes and increased frost resistance in both adapted and non-adapted plants to low temperatures [14]. Expression of the Arabidopsis *CBF1* gene in tomatoes has also been shown to be involved in cold tolerance [15]. The role of CBF orthologous genes in abiotic stress tolerance has been identified in a wide range of model and other crop plants such as Arabidopsis [16], rapeseed [17], rice [18], maize [19], wheat [20], barley [21], tomato [22], tobacco [23], cotton [24], and *Capsella bursa-pastoris* [25]. The widespread presence of the *DREB1*/*CBF* regulatory system and their association with stress tolerance in plants make them a suitable biomarker system for studying cold stress conditions [26]. The cold-responsive (COR) genes as CBF targets are the last players of the ICE-CBF-COR cascade, with their important role in cold stress tolerances [27]. Cold acclimation or priming with low temperatures has been showing positive results in adaptation and stress tolerance [28]. A comprehensive review of genetics and physiological changes in cold stress in plants has been published recently in which the complex processes of cold stress tolerance are summarized and well addressed [29].

The cold stress and unexpected low temperatures are causing significant damage to agricultural production all over the world [30]. These damages can be assessed by investigating the physiological and phytochemical changes in stressed plants. Meanwhile, one of the fundamental prerequisites to dealing with freezing stress is to introduce tolerant cultivars. This can be facilitated by studying and learning the mechanisms of stress responses in tolerant plants. Therefore, this study was conducted to assess the effects of freezing stress on cold-acclimated and non-acclimated winter and spring biotypes of camelina at the phytochemical and molecular levels to test the hypothesis that cold-acclimatized lines may exhibit more tolerance to freezing stress due to the pre-activation of molecular and physiological processes involved in low-temperature adaptation.

## 2. Results

The following results show the effect of freezing stress (−5 °C) on a freezing-tolerant (FT) and a freezing-sensitive (FS) biotype of Camelina plants with cold acclimation (AC) and without cold acclimation (NA) before stress treatment.

### 2.1. Biochemical Assessments

**Figure 1:**
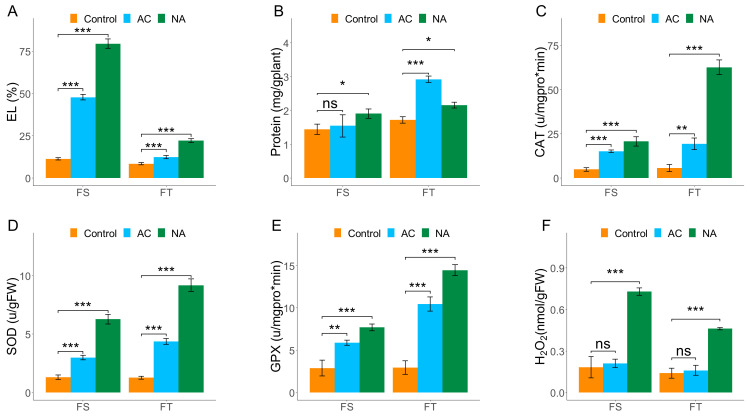

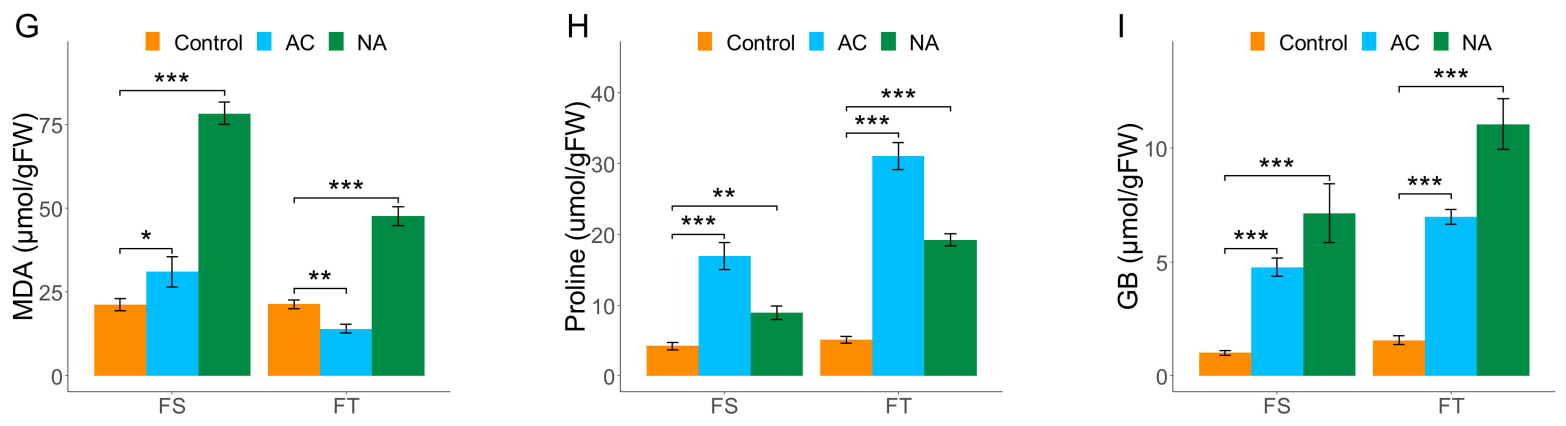
The effect of freezing stress on biochemical properties of two Camelina biotypes (freezing sensitive (FS) and freezing tolerant (FT)) with (AC) and without cold acclimation (NA). EL: electrolyte leakage; CAT: catalase; SOD: superoxide dismutase; GPX: guaiacol peroxidase; MDA: malondialdehyde; GB: glycine betaine. The ns, *, **, and *** show non-significant differences or significant differences at *p* ≤ 5%, 1%, and 0.1%, respectively.

The EL determination assay was successfully applied to verify the tolerance degree of the FT and FS genotypes of Camelina. The EL significantly (*p* < 0.001) increased in FT and FS Camelina DH lines after freezing stress when compared to their controls. However, the EL percentage was lower in the FT line and in cold-acclimated (AC) treatment (Figure 1a). Less EL indicates higher cellular membrane stability and freezing tolerance. A comparison of the protein content in the Camelina biotypes showed that the soluble proteins in the FT line after the acclimation condition were significantly more than that of in control and the non-acclimated plants (Figure 1b). The catalase enzyme (CAT) activity increased significantly after freezing stress, with its highest level being detected in the non-acclimated FT Camelina biotype. (Figure 1c). Interestingly, similar findings were observed for the superoxide dismutase (SOD) and guaiacol peroxidase (GPX), where both enzymes’ activity was induced by freezing stress (Figure 1d,e). The highest hydrogen peroxide (H_2_O_2_) and malondialdehyde (MDA) contents were recorded in samples of non-acclimated plants of both FT ad FS lines after freezing stress (Figure 1f,g). However, the H_2_O_2_ and MDA levels in the FS biotype were substantially more in comparison to the FT line (Figure 1f,g). Furthermore, proline content was also increased in both FS and FT biotypes of Camelina exposed to freezing stress. However, the acclimation for two days before the freezing stress resulted in higher proline content in comparison to the non-acclimated plants (Figure 1h). The content of glycine betaine (GB) was also significantly increased when both lines were exposed to freezing stress. The acclimation treatment did now show any significant effect on the GB content of stressed plants in comparison to their non-stressed counterparts (Figure 1i).

### 2.2. Expression Profiling of ICE, CBF, and COR Genes

**Figure 2:**
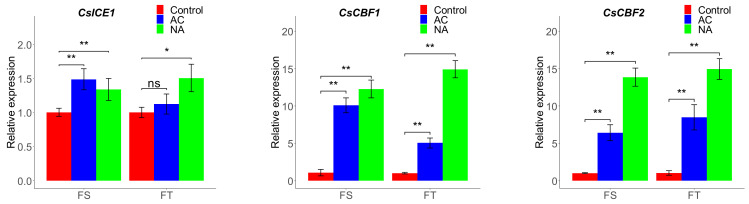

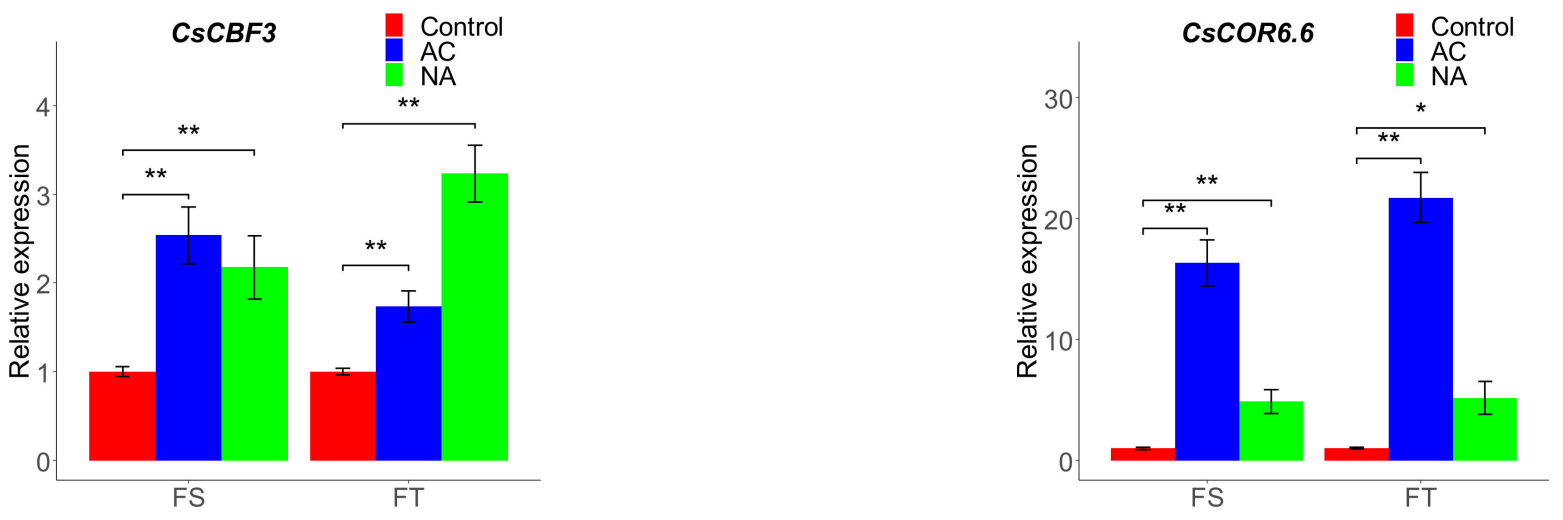
The *CsICE1*, *CsCBF1*, *CsCBF2*, *CsCBF3*, and *CsCOR6.6* genes in two Camelina biotypes (freezing sensitive (FS) and freezing tolerant (FT)) with (AC) and without cold acclimation (NA) after freezing stress. The ns, *, **, and *** show non-significant differences or significant differences at *p* ≤ 5%, 1%, and 0.1%, respectively.

A set of selected genes from the *ICE*-*CBF*-*COR* pathway with their confirmed association with freezing tolerance (in Arabidopsis and other plants) were investigated for their relative expression upon freezing stress in cold-acclimated (AC) and non-acclimated (NA) Camelina biotypes by real-time quantitative PCR. The expression pattern of *CsICE1*, *CsCBF1*, *CsCBF2*, *CsCBF3*, and *CsCOR6.6* genes in the freezing-sensitive (FS) and freezing-tolerant (FT) biotypes are shown in Figure 2. A significantly higher (*p* < 0.05) expression of the *CsICE1* gene was detected in both biotypes after exposure to freezing stress (Figure 2a). However, the *CsICE1* expression level in the acclimated (AC) FT biotype was not statistically significant (*p* < 0.05) when compared to the control. The expression of all CBF genes was induced in both biotypes exposed to freezing stress (Figure 2). The FS biotype showed more induction of *CsCBF1* and *CsCBF3* than the FT biotype in acclimated samples, which was quite the opposite in the case of the *CsCBF2* expression pattern. Both biotypes had their maximum expression of all CBF genes after freezing stress and in non-acclimated samples at levels significantly higher than the controls (*p* < 0.01). Interestingly, the *CsCOR6.6* gene in the two contrasting Camelina biotypes showed its maximum expression level in cold-acclimated samples exposed to freezing stress (Figure 2). Even though the expression of the COR gene in non-acclimated samples was significantly higher than its level in the control plant, the relatively higher values in AC-treated samples indicate the inducibility of this gene upon cold acclimation rather than the direct freezing stress.

### 2.3. Identification of Syntelogs and Gene Duplication Analysis

Since *C. sativa* is an allohexaploid plant, there is more than one copy of the selected *ICE-CBF-COR* genes in Camelina in comparison to the one copy number in closely related plant species *A. thaliana* genome. We identified three copies of *CsICE1*, *CsCBF2*, *CsCBF3*, and *CsCOR6*.6 genes and only two copies of *CsCBF1* in the Camelina genome (Table 1). Arabidopsis *AtCBF1-3* genes are located on chromosome number 4, whereas the *CsCBF1* and *CsCBF3* genes in the Camelina genome are distributed on chromosomes number 10, 11, and 12 and *CsCBF2* discovered on chromosomes 10 and 12 only. The *AtICE1* was found on chromosome number 3 in Arabidopsis but on chromosomes 4, 6, and 9 in Camelina. The *AtCOR6.6* gene in Arabidopsis was on chromosome 5, and chromosomes 8, 13, and 20 in Camelina.

**Figure 3:**
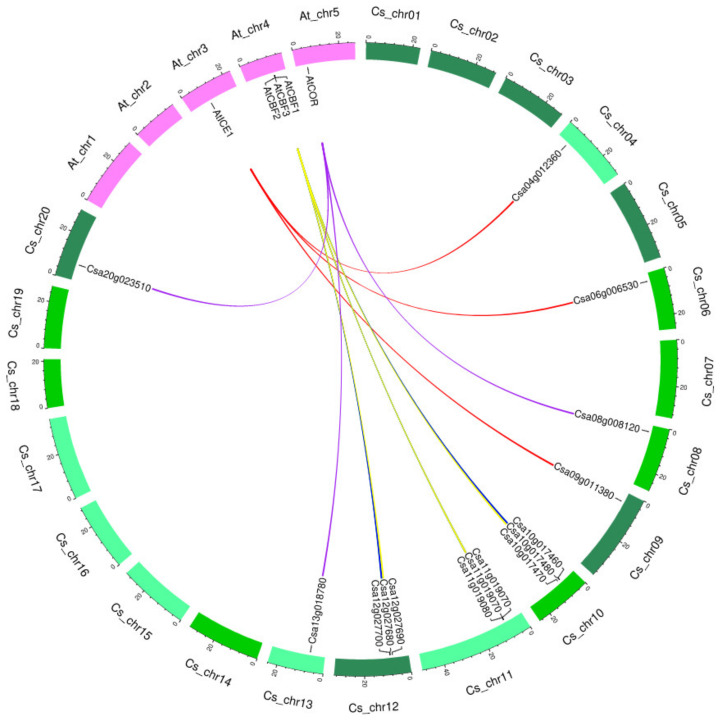
Synteny analysis of the selected *ICE-CBF-COR* genes in *A. thaliana* and *C. sativa*. The At_chr and Cs_chr show the chromosome number in *A. thaliana* and *C. sativa*, respectively. Three sub-genomes of C. sativa represent in different green colors.

The synteny of the selected *ICE-CBF-COR* genes in *A. thaliana* and *C. sativa* is represented in Figure 3. Synteny analysis of the *CsICE1*, *CsCBF1*, *CsCBF2*, *CsCBF3*, and *CsCOR6.6* genes in comparison to *A. thaliana* genome showed that gene copies of *C. sativa* were located on G1, G2, and G3 sub-genomes of *C. sativa* (presented in different green colors in Figure 3). For example, *AtICE1*, which is located on chromosome 4 in *A. thaliana*, was detected on chromosomes 4 (G1), 6 (G2), and 9 (G3) of *C. sativa*, reflecting the more copy numbers of these genes as a result of gene duplications.

## 3. Discussion

Freezing stress is one of the main severe environmental factors affecting the growth and yield of crops and a major limiting factor in introducing new crops/cultivars around the world. Camelina is a re-emerging oilseed crop with the potential to grow in a wide range of climates as a winter or spring crop [31]. Accordingly, there are two biotypes of Camelina plants (e.g., spring and winter biotypes) with different responses to low temperatures [5,32], which are categorized by morphology [33] and/or allele-specific molecular markers [34]. The winter biotype of Camelina is typically known as freezing tolerant (FT), and the spring/summer biotype is commonly referred to as freezing sensitive (FS). The two biotypes are equipped with different mechanisms to cope with or/and respond to low temperatures [32]. Among the biomarkers to assess the freezing tolerance in various plant species, electrolyte leakage (EL) quantification is a common and reliable method to estimate freezing tolerance in plant species [35]. A favorable freezing tolerance was observed in Camelina seedlings with acclimation treatment due to a lower level of electrolyte leakage (EL). The significantly higher and cold stress-responsive EL rate in FS Camelina biotype in comparison to FT can be considered a decisive factor in screening studies to identify tolerant lines [36]. The lower EL level in cold-acclimated seedlings can correspond to the lower level of damage in freezing-stressed plants, reflecting the activation of defensive factors, including biochemical and transcriptomic responses. Changes in the structure and function of cell membranes are the first effects of stress and often are related to oxidative damage. Plants produce a series of antioxidant systems that play their role in detoxifying reactive oxygen species (ROS) [37]. The relatively lower antioxidant enzymes activity of catalase (CAT), superoxide dismutase (SOD), guaiacol peroxidase (GPX), and glycine betaine (GB) in cold-acclimated seedlings, compared to their non-acclimated counterparts, indicates the reduced extent of the cell damages or need for their activities in freezing-stressed Camelina plants. Antioxidant enzymes also play a key role during freezing stress to avoid the accumulation of hydrogen peroxide [38]. As expected, the H_2_O_2_ level was significantly lower in cold-acclimated samples in our study. The role of glycine betaine (GB) in freezing tolerance was investigated years ago in Arabidopsis [39]. In our samples, we could not find considerable patterns in GB content to be recognized among the contrasting biotypes.

The deteriorating effect of low temperatures on membrane structure and the consequent water imbalance in plant tissues are long known [40]. Plant cells can sense cold stress by altering membrane fluidity [41]. After the sensation of cold temperatures by plants, numerous signals, such as Ca^2+^, ROS, abscisic acid, salicylic acid, and other phytohormones, are generated and released [42]. These may initiate the induction or the regulation of several genes’ expressions. Understanding the gene expression under stress conditions can provide a better fundamental insight into environmental stress resilience in plants. The activated signals can influence the expression pattern of various genes, such as protein kinase, transcription factor, and *COR* genes, as well as their subsequent physiological activities [41].

Low temperatures significantly alter the *ICE*/*CBF*/*COR* signaling pathway, including *inducer of CBF expression* (*ICE*), *C-repeat binding factor* (*CBF*), and *cold-regulated* (*COR*) genes, which play a significant role in freezing sensing and plants responses to cold stress [27]. In Arabidopsis, there are three *CBF* genes, including *CBF1*, *CBF2*, and *CBF3* (also known as *DREB1B*, *DREB1C*, and *DREB1A*, respectively) that induce the low-temperature response signaling pathway [41]. Plant *CBF* genes are involved in cold tolerance by inducing the expression of downstream genes, such as *COR* genes, through metabolic changes and physiological processes [43,44,45]. *CBF* genes themselves are regulated by other transcription factors, including *ICE1*, *MYB15*, and *CAMTA3* [46,47]. The *ICE*-*CBF*-*COR* signaling cascade is one of the most well-known transcriptionally regulated pathways of Arabidopsis in response to cold stress, involving various genes, including *COR15A*, *COR15B*, *COR47*, and so on [48]. In our study, relative expression of the *CsICE1* gene in Camelina biotypes was slightly but significantly induced by freezing stress, indicating its possible involvement in chilling tolerance in the Camelina plant. Anderson et al. [49] reported that *CBF1/2* is an upstream regulator of *GOLS3* and *COR15A* genes, which are participants of ROS scavenging processes in stressed Camelina guard cells. Horvath et al. [50] claimed that *CBF* gene expression was induced in both spring and winter in Camelina biotypes under freezing stress and, therefore, not responsible for the freezing tolerance in the camelina winter biotypes.

It was also noticed that *CBF1*, *CBF12*, *CBF13*, and *ICE1* genes are reported to be induced in Arabidopsis for only a short time (15 min) under cold stress conditions [51]. On the other hand, Wang et al. [32] reported the upregulation of *CBF* genes in a winter biotype of Camelina (named Joelle), which was somewhat similar to our results, where the expression of all three CBF genes was highest in FT (Winter biotype) after frost stress. As stated by Wang et al. [32], freezing tolerance in Camelina biotypes is complicated when considering the cold acclimation and vernalization processes, especially in winter biotypes.

In the synteny analysis, it was found that there were three copies for most of the studied genes in Camelina, and it may be a reason for the higher freezing tolerance of Camelina (a hexaploid plant) rather than *A. thaliana* (a diploid plant) in general. Distribution of *CsICE1*, *CsCBF1*, *CsCBF2*, *CsCBF3*, and *CsCOR6.6* genes on *C. sativa* and *A. thaliana* chromosomes confirmed hexaploidy of Camelina and revealed orthologous relationships between these two closely related plant species.

Furthermore, our previous genetic analysis [5] indicated that freezing tolerance in Camelina is rather controlled by additive effects of genes. The finding of this study and the results of Wang et al. [32] indicated that there may be different pathways/genes involved in cold acclimation-induced freezing tolerance in Camelina. In the current study, we assessed the overall expression of the existing selected genes on the Camelina genome. It may be of high interest to investigate the expression of each copy of the homologous genes separately in future studies to find out if their different positions on different chromosomes may influence their expression inducibility. The genome–environment associations and more innovative approaches, such as genomic estimated adaptive value models, may shed more light on predicting stress tolerance in crops such as Camelina [52,53,54,55]. Further studies on genetic variations in the freezing tolerance of camelina biotypes can lead to developing freezing-related SNP markers in the winter biotype of camelina for rapid screening of new breeding lines.

## 4. Materials and Methods

### 4.1. Plant Materials and Experimental Treatments

**Figure 4:**
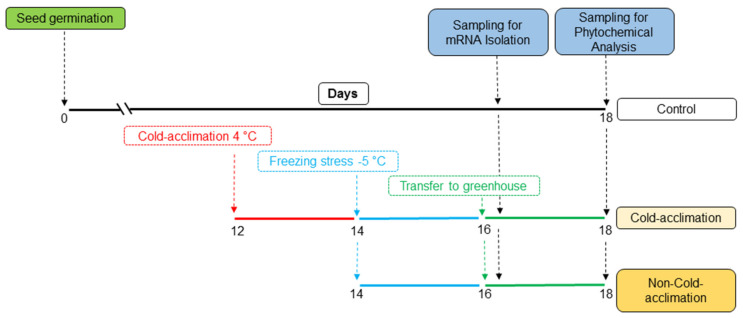
The schematic diagram illustrating the experiment and treatment design.

Based on substantial screening test results among the several doubled-haploid lines, DH8 and DH34 lines with low and high tolerance to freezing stress, respectively, were selected [5,34]. The selected lines were subjected to two pre-treatments, including cold acclimation and non-acclimation, prior to freezing stress, along with controls in triplicates. The schematic diagram of the experimental design is presented in Figure 4. Seeds were germinated in peat moss-containing pots (↔ 8 cm × ↕ 10 cm) in a temperature-controlled greenhouse with day/night temperatures of 22/18 °C, respectively (five seedlings were kept in every pot and considered as one biological replicate). The cold-acclimation treatment (4 °C) was started on day 12 after germination and for two days in a temperature-controlled phytotron growth chamber (Conviron E-15; Conviron Controlled Environments Ltd., Winnipeg, Canada) with similar photoperiod and light intensity. The freezing stress (48 h at −5 °C) was applied to both cold-acclimated and non-cold-acclimated plants by placing them in a freezer device (JTUL150, Jal Tajhiz Co, Iran) under short-day condition (8 h light/16 h dark) at day 16 after germination. The treated and control plants were subjected to sampling on days 16 and 18 after germination (Figure 4). The instantly frozen samples in liquid N were kept at −80 and −20 °C for RNA extraction and biochemical analyses, respectively.

### 4.2. Electrolyte Leakage (EL)

The EL value was assayed according to the original method of Kim et al. [6], with modifications adopted in the screening experiment [36].

### 4.3. Preparation of Enzyme Extracts and Antioxidant Enzymes Activity

The plant extracts were obtained by grinding 0.4 g of frozen leaf samples in liquid nitrogen in a mortar and pestle to a fine powder. The powdered specimens were then transferred into 2 mL Eppendorf tubes to which 1800 μL of 0.1 M phosphate buffer (pH 7.0) containing 0.1 M EDTA was added, briefly vortexed, and centrifuged for 15 min at 14,000 rpm at 4 °C. The supernatant was transferred to clean Eppendorf tubes and stored on ice for the enzyme activity assays [56].

The catalase (CAT; EC: 1.11.1.6) enzyme activity was measured according to the method of Chance and Maehly [57]. Concisely, the 3 mL reaction mixture contained 10 mM H_2_O_2_, 50 mM potassium phosphate buffer (pH 7.0), and 100 μL of enzyme extracts. The decomposition of H_2_O_2_ was recorded at 240 nm. The results were expressed as EU (μM of H_2_O_2_ decomposed per minute) mg^−1^ protein.

For superoxide dismutase (SOD; EC 1.15.1.1) activity, a method described by Giannopolitis and Ries [58] was applied. The reaction mixture contained 50 mM potassium phosphate buffer (pH 7.8), 12 μM methionine, 75 μM p-nitro blue tetrazolium chloride (NBT), 1 μM riboflavin, and 300 mL of enzyme extract. One unit of SOD activity was defined as the amount of enzyme required to obtain a 50% inhibition rate of NBT reduction that was recorded at 560 nm, and SOD activity was reported as enzyme unit per mg protein.

Guaiacol peroxidase (GPX; EC:1.11.1.9) activity was quantified according to the Chance and Maehly method [51]. The 3 mL reaction solution contained 50 mM phosphate buffer (pH 7.0), 10 mM H_2_O_2_, 20 mM guaiacol, and 600 μL of enzyme extract. The change in absorbance at 470 nm was recorded for 1 min.

### 4.4. Total Soluble Protein

To estimate the total protein content of the samples, 0.1 mL of enzyme extract was mixed with 4.9 mL of Bradford reagent and incubated for 15 min after gentle vortexing. The absorbance was read at 595 nm using a spectrophotometer (1800 UV–VIS, Shimadzu Inc., Kyoto, Japan) in triplicates. Bradford solution without the extract was used as blank. Serum bovine albumin (BSA) was used as a standard protein (0, 4, 8, 12, 16, and 20 μL) to establish the calibration curve and quantification [59].

### 4.5. Glycine Betaine (GB)

The glycine betaine was assayed following the Grieve and Grattan [60] method. First, 250 mg of leaf tissue was ground and mixed with 10 mL of distilled water. After filtration, 1 mL extract was mixed with 1 mL sulfuric acid. A 0.5 mL of this mixture was mixed with 0.2 mL potassium tri-iodide solution and then cooled in an ice bath for 16 h. The organic layer was centrifuged at 10,000 rpm for 10 min at 0 °C. Two ml of ice-cold distilled water and 20 mL 1,2-dichloromethane were added to the mixture, and absorbance was measured at 365 nm after 2 h. The GB concentration was calculated using a standard curve and expressed in μM g^−1^ fresh weight of the leaf.

### 4.6. Proline Content

Proline concentration was quantified by following the method of Bates et al. [61]. Fresh plant leaf samples (0.5 g) were homogenized in a chilled mortar and pestle with three ml of 5-sulfosalicylic acid (3%). Leaf extract (2 mL) was gently mixed with 2 mL of acid ninhydrin and 2 mL of glacial acetic acid in test tubes before incubation for 1 h in hot water (100 °C). Toluene (4 mL) was added to the test tubes filled with the reaction mixture and vigorously shaken for 15–20 s. The absorbance was recorded at 520 nm, and proline content was calculated using a standard curve.

### 4.7. Hydrogen Peroxide (H_2_O_2_)

The H_2_O_2_ content of the leaves was measured spectrophotometrically at 560 nm following the colorimetric reaction [62]. In brief, 0.25 g of plant leaf samples were homogenized in 1 mL of 10% phosphoric acid, and the supernatant was used for the quantification of H_2_O_2_. Sample extracts (50 µL) were mixed with a reaction mixture (950 µL) containing 100 µM Xylenol Orange, 250 µM ammonium ferrous sulfate, 100 µM sorbitol, and 25 µM sulfuric acid. Different concentrations of H_2_O_2_ (0.25–10 µM) were used to draw the calibration curve, and the results were expressed as µM g^−1^ FW.

### 4.8. Estimation of Lipid Peroxidation (MDA)

Malondialdehyde (MDA), a biomarker of lipid peroxidation, was quantified by thiobarbituric acid (TBA) assay following the original method of Heath and Packer [63]. Leaf samples of 0.5 g were extracted with 2 mL of 0.1% trichloroacetic acid (TCA) in a cold mortar with a pestle. To stop further peroxidation, 20% butylated hydroxytoluene in absolute ethanol (40 μL) was added to the solution [64] before vortexing and centrifugation at 15,000 rpm (15 min at 4 °C). Supernatant (0.25 mL) was added to 20% TCA (1 mL) containing 0.5% TBA, mixed and centrifuged for 5 s before incubation for 30 min at 96 °C. The reaction was terminated by cooling on ice and centrifugation at 8000 rpm (3 min). To calculate the MDA concentration (nM g^−1^ fresh weight (FW)), non-specific absorption at 600 nm was subtracted from the absorption at 532 nm by using the absorbance coefficient (156 mM^−1^ cm^−1^) of extinction.

### 4.9. Total RNA Extraction and cDNA Synthesis

Total RNA was extracted by a CTAB-based protocol [65] from deep frozen leaves of two-week-old camelina seedlings after grinding to a fine powder in N_2_. RNA quantity was measured in a NanoDrop 1000 spectrophotometer (Thermo Fisher Scientific, Waltham, MA, USA) at 260 nm. The RNA integrity was assessed on an EcoSafe-stained 1% agarose gel after treating the samples with DNase I enzyme (Thermo Fisher Scientific). Complimentary DNAs were produced by reverse transcription of total RNA (5 μg) as a template and M-MuLV RT enzyme supplied in Maxima Reverse Transcriptase kit (Thermo Fisher Scientific) with oligo (dT)_20_ primers according to the manufacturer’s guidelines. Primers of selected camelina freezing tolerance genes and a control *ef1* housekeeping gene (Table S1) were tested by PCR amplification using Go Taq DNA polymerase (Promega, Madison, WI, USA). PCR products with expected sizes were visualized on 1.5% (*w*/*v*) ethidium bromide-stained agarose gel in 1 × TBE buffer.

### 4.10. Real-Time PCR Conditions (RT-qPCR) and Gene Expression Analysis

Quantitative Real-Time PCR reactions were performed using SYBR Green I technology in a C1000 ™ Thermal Cycler (Bio-Rad, Hercules, CA, USA) using Maxima SYBR Green/ROX qPCR Master Mix (Thermo Fisher Scientific, Cat. No: K0221) in 96-well low-profile optical plates. The final volume of the qPCR reaction was 10 μL, including 1 μL of cDNA, 4 μL of Mater Mix, 0.5 μL (100 μM) of F&R primers, and 4 μL of PCR-grade water. A melting curve analysis was conducted (65–95 °C) at the end of PCR reactions to confirm the PCR product specificity. The PCR efficiency was determined and approved based on the Cq values of the standard dilutions of cDNAs for all primer pairs. A camelina *ef1* gene was used as endogenous control after its stability was tested and approved for normality of residuals (Shapiro–Wilk’s test) and homogeneity of variances (Bartlett’s test) using R-Studio software (Version 3.5.1.) [66].

### 4.11. Synteny Analysis

The *ICE1*, *CBF1*, *CBF2*, *CBF3*, and *COR6.6* paralogous information, including chromosomes location, the sequence, and the size of the genes, were retrieved from Ensembl Plants [67] using BioMart and CamRegBase; http://camregbase.org/ accessed on 1 March 2022 [68] database, then analyzed and drawn in shinyCircos R/Shiny software environment [69].

### 4.12. Statistical Analysis

The biochemical data were analyzed by Student’s *t*-test using R 4.1.2 (accessed on 1 March 2022) [70] and shown as mean values with standard deviations (±SD) among three biological replicates. Gene expression data were presented as fold changes of the examined genes calculated by the 2^−∆∆Ct^ method [71].

## 5. Conclusions

The re-emerging and important camelina oilseed plant is potent for breeding toward cold-resistant cultivars, especially when winter biotypes have already been developed and cultivated. Our results based on the biochemical and transcriptome analyses indicated that the low-temperature acclimation prior to frost stress can significantly alter and rather enhance the performance of plants upon exposure to freezing stress. This became evident when almost all stress-associated biomarkers declined in cold-acclimated plants when compared to the non-acclimated ones upon stress treatment. The investigated candidate genes of Camelina under cold-acclimated and non-acclimated conditions demonstrate the role of the ICE-CBF-COR pathway in the freezing tolerance of Camelina. The results of this study indicate the importance of plant acclimation at low temperatures prior to freezing stress and reveal the capability of Camelina biotypes to cope with unfavorable environmental conditions. These results may be useful in genetic engineering and breeding programs to utilize the components of the important ICE-CBF-COR pathway in freezing tolerance objectives in connection to Camelina breeding.

## Figures and Tables

**Table 1:** Comparison of the selected *ICE-CBF-COR* genes in *A. thaliana* and *C. sativa*.

Gene Name	At Gene Stable ID		Chr (*n* = 5)	Length bp (aa)	Cs Gene Stable ID	Chr (*n* = 20)	Length bp (aa)	E-Value	Identity (%)
*ICE1*	*AT3G26744*	3		2612 (494)	*Csa09g011380*	9	1497 (255)	4.67 × 10^−3^	96.64
					*Csa06g006530*	6	2171 (499)	0	93.79
					*Csa04g012360*	4	2581 (349)	0	93.57
*CBF1 (CsDREB1b)*	*AT4G25490*	4		1216 (213)	*Csa10g017460*	10	1218 (212)	3.33 × 10^−127^	85.85
					*Csa12g027680*	12	929 (211)	3.27 × 10^−127^	86.26
					*NA*	-	-	-	-
*CBF2 (CsDREB1c)*	*AT4G25470*	4		985 (216)	*Csa11g019080*	11	2540 (217)	1.37 × 10^−71^	53.16
					*Csa10g017480*	10	1008 (218)	1.11 × 10^−132^	83.49
					*Csa12g027700*	12	1008 (217)	8.46 × 10^−94^	80.77
*CBF3 (CsDREB1a)*	*AT4G25480*	4		1390 (216)	*Csa12g027690*	12	995 (218)	6.83 × 10^−129^	84.16
					*Csa11g019070*	11	1093 (129)	7.78 × 10^−97^	85.15
					*Csa10g017470*	10	953 (218)	6.33 × 10^−130^	83.27
*COR6.6*	*AT5G15970*	5		1024 (66)	*Csa08g008120*	8	1428 (66)	9.19 × 10^−24^	93.94
					*Csa13g018780*	13	1619 (66)	9.19 × 10^−24^	93.94
					*Csa20g023510*	20	845 (66)	3.14 × 10^−23^	92.42

## Supplementary Materials

The following supporting information can be downloaded at: https://www.mdpi.com/article/10.3390/plants11223178/s1, Table S1: Target genes and oligonucleotide primers applied in RT-qPCR.
