# Supplementary material for: Biochemical and Transcriptional Responses in Cold-Acclimated and Non-Acclimated Contrasting Camelina Biotypes under Freezing Stress
Source: Plants (Basel). 2022 Nov 21;11(22):3178. doi: 10.3390/plants11223178 (PMC9693809; doi:10.3390/plants11223178)
Supplement: Supplementary file 1 [file plants-11-03178-s001.zip › plants-1995236-supplementary.pdf]

Supplementary Materials

Table S1: Target genes and oligonucleotide primers applied in RT-qPCR.

Gene	Accession numbers	Forward primer (5'-3')	Reverse primer (5'-3')	Amplicon size (bp)
CsICE1	LOC104715011<br>LOC104779889<br>LOC104790312	AGCTTCCATCCATTAACGCCT	ACTCTTGCTTGCTGGCCTT	104
CsCBF1 (CsDREB1b)	LOC104730883<br>LOC104722420<br>LOC104717716	AGGAGGCAATGTTCGGGATG	TCTCCCTCGCCGTCATAATTG	106
CsCBF2 (CsDREB1c)	LOC104722422	CGTTTGGGATAAGAAAGGTTTACCA	TCCACGCTTCTTCACTGGAA	90
CsCBF3 (CsDREB1a)	LOC104730886<br>LOC104722423<br>LOC104717717	CGGCTCCGATTACGAGTCTT	GTGACGGGTCTCACGAAACT	115
CsCOR6.6	LOC104705885<br>LOC109128505<br>LOC109131125	GCTGGCAAAGCTGAGGAGAA	TTTCCAGCTTGTTGAGCGGA	92
Csef1	LOC104782387<br>LOC104737839<br>LOC104737777	GTACCCACCATTGGGACGTT	TTCTTGACTGCAGCCTTGGT	123
